# Exploration of probiotic attributes in lactic acid bacteria isolated from fermented *Theobroma cacao L.* fruit using *in vitro* techniques

**DOI:** 10.3389/fmicb.2023.1274636

**Published:** 2023-09-21

**Authors:** Mausamy C. Nandha, Rachana M. Shukla

**Affiliations:** ^1^Department of Microbiology and Biotechnology, School of Science, Gujarat University, Ahmedabad, India; ^2^Department of Microbiology, Gandhinagar Institute of Technology, Gandhinagar, India

**Keywords:** probiotic attributes, antibiotic sensitivity, simulated gastrointestinal digestion, *Theobroma cacao L*., lactic acid bacteria

## Abstract

Probiotics are known for their health-promoting properties and are recognized as beneficial microorganisms. The current investigation delves into the isolation and comprehensive *in vitro* characterization of lactic acid bacteria (LAB) obtained from the Indian-origin *Theobroma cacao L.* Forastero variety to assess their potential as probiotic candidates. Eleven LAB isolates were obtained, and among them, five exhibited classical LAB traits. These five isolates underwent rigorous *in vitro* characterization to evaluate their suitability as probiotics. The assessments included resilience against acid and bile salts, which are crucial for probiotic viability. Additionally, the isolates were subjected to simulated gastric and pancreatic fluids and lysozyme exposure to assess their survival rates. Auto- aggregation, co-aggregation, hydrophobicity, and exopolysaccharide production were also examined. The inhibitory potential of α-glucosidase, an enzyme related to glucose metabolism, was measured, and antioxidant activity was evaluated using DPPH and ABTS assays. A safety assessment was conducted to confirm the non-pathogenic nature of the isolates. Among the five isolates, CR2 emerged as a standout candidate with maximal bile salt hydrolase activity, phenol resistance, and lysozyme resistance. CR2 and CYF3 exhibited notable survival rates under simulated conditions. The isolates displayed variable degrees of auto-aggregation, co-aggregation, and hydrophobicity. CR2 exhibited the highest exopolysaccharide production (0.66 mg/mL), suggesting diverse applications in the food industry. CR2 also demonstrated the highest inhibition rate against α-glucosidase (56.55%) and substantial antioxidant activity (79.62% DPPH, 83.45% ABTS). Safety assessment confirmed the non- pathogenic nature of the isolates. Molecular characterization identified CR2 as *Lactococcus lactis* subsp. *lactis* and CYF3 as *Limnosilactobacillus fermentum*. Both strains exhibited commendable probiotic and technological attributes, positioning them as promising candidates for functional foods and beyond. This study provides valuable insights into the *in vitro* characterization of LAB isolated from Indian *Theobroma cacao L.*, highlighting their potential as probiotic candidates with advantageous traits, including survival in hostile conditions, beneficial enzymatic activities, bioactivity, and other essential attributes.

## 1. Introduction

The microbiota inhabiting the gastrointestinal tract, primarily concentrated in the intestinal region, engages in a mutually beneficial relationship with the host organism. The term ‘normobiosis’ characterizes the condition of the intestinal ‘ecosystem’ where health-promoting microorganisms predominate a multitude of potentially adverse microorganisms. Conversely, ‘dysbiosis’ pertains to a situation in which one or a minor portion of potentially harmful microorganisms exert dominance, thereby engendering a state of pathological imbalance (Roberfroid et al., 2010). The inhabited and productive microbial communities found in these habitats are often designated as probiotics. These microorganisms perform essential metabolic, immunological, and protective functions that play a critical role in promoting the health of both animals and humans (Jumpertz et al., 2011; Wang et al., 2011; Goldsmith and Sartor, 2014). Diverse probiotic strains potentially serve as reservoirs of numerous metabolites that actively contribute to the stability of the genome, intercellular communication, and epigenetic regulation. These attributes render them compelling contenders for integration into the human dietary framework (Pihurov et al., 2021). The utilization of starter cultures has evolved into a cutting-edge practice across the majority of industrial food fermentation processes. This adoption enables the standardization of processes, mitigation of hygiene-related risks, and facilitation of product diversification.

Cocoa, derived from the *Theobroma cacao L.* tree indigenous to the Amazon basin and tropical region of South and Central America South America, is vital for producing chocolate. Within the cocoa bean, consisting of paired cotyledons and an embryo encased in a protective seed coat, lies a sweet and pale mucilaginous pulp (Figueroa-Hernández et al., 2019). As cocoa matures, the pulp’s fructose and glucose levels rise, while it also contains pectin, various polysaccharides, proteins, amino acids, minerals, vitamins, and citric acid (Camu et al., 2008; Lima et al., 2011; De Vuyst and Weckx, 2016). Once cocoa beans are extracted from pods, a natural fermentation process begins, primarily guided by yeast, lactic acid bacteria (LAB), and acetic acid bacteria (AAB). This microbial activity generates diverse metabolites that diffuse into the cocoa seeds (Schwan and Wheals, 2004; Camu et al., 2007). These biochemical changes within the seeds lead to reduced bitterness and astringency, ultimately preventing embryo germination (Kadow et al., 2013, 2015). Consequently, fermenting cocoa beans becomes a critical postharvest stage significantly shaping chocolate quality.

Among these practices, the genus *Lactococcus*, classified as a mesophilic lactic acid bacterium, stands out as the predominant starter bacteria in the dairy sector. It finds extensive application in the production of diverse fermented foods, including cheese varieties, cultured milk products, cream buttermilk, and other established fermented dairy commodities (Tamang et al., 2020). Furthermore, these microorganisms exhibit the capacity to metabolize a diverse array of substrates, thrive effectively under microaerophilic or anaerobic environments, generate various acids (such as lactate, acetate, and propionate), and influence the reduction in pH and redox potential. In addition, they synthesize bacteriocins and antifungal agents, which demonstrate activity against a wide or delimited spectrum of specific target entities. These encompass both agents of food spoilage and toxigenic microorganisms. Moreover, these microorganisms contribute to the augmentation of quality attributes and the inherent preservation mechanisms within fermented food products (Gänzle, 2009). While considering the identification of novel probiotic microorganisms, ensuring their safety remains a paramount concern. Fortunately, *Lactococcus* sp. do not raise safety concerns due to their similarity to *Lactobacillus* sp. in possessing Foods for Specified Health Use (FOSHU) recognition, Generally Recognized as Safe (GRAS) designation, and Qualified Presumption of Safety (QPS) classification (Yamaguchi, 2003; Kimoto et al., 2007). However, a multitude of newly discovered *Lactococcus* species, sourced from plant materials, have exhibited diverse technological advantages. These species have the capacity to produce organic acids and aromatic compounds, which hold the potential to enhance the efficacy of the evaluated products (Rodríguez et al., 2019).

Prior investigations have effectively isolated and characterized LAB originating from *Theobroma cacao L*. across various geographical locations. Noteworthy examples encompass the identification of *Lactobacillus fermentum* TcUESC01 and *Lactobacillus plantarum* TcUESC02 through the natural fermentation process of cocoa beans. These strains, identified as potential probiotic contenders, were previously isolated and analyzed (Saito et al., 2014; Melo et al., 2017; Oliveira et al., 2018). Remarkably, the isolation of LAB strains with a preference for fructose-rich environments is evident. Strains such as *Pediococcus acidilactici, Lactobacillus plantarum, Pediococcus pentosaceus, Bacillus subtilis*, and *Leuconostoc pseudomesenteroides* have been successfully extracted from the region of Bahia State, Brazil (Viesser et al., 2020). Furthermore, similar investigations have been undertaken in locations like Tomé-Açu in Pará, as well as in the context of cocoa samples procured from Bahia, Brazil, and the Brazilian Amazon. These strains, well-adapted to environments abundant in fructose, exhibit substantial potential for incorporation into probiotic formulations (Chagas Junior et al., 2021, 2022). Notably, the aforementioned LAB strains were previously identified as prospects for probiotic utilization in earlier research endeavors. This collective body of work showcases the valuable diversity and adaptability of LAB strains sourced from cocoa and its diverse locales, underscoring their potential significance in advancing probiotic applications.

In the context of India, the cultivation of *Theobroma cacao L*. is notably concentrated in states such as Kerala, Tamil Nadu, and Karnataka. This agricultural pursuit has also expanded to encompass a diverse range of locales, including urban centers like Navsari in Gujarat, as well as several other regions within the state, including Gir-Somnath, Junagadh, Bhavnagar, and Kutch. A distinctive attribute characterizing these regions is the prevalence of coconut trees or palm orchards, a factor significantly influencing the course of this expansion. However, it’s worth noting that despite this extensive agricultural activity, the isolation and systematic characterization of LAB from the Navsari region of India have yet to be documented. Particularly within the realm of cocoa production, the distinctive fermentation process employed in the Navsari region of Gujarat remains a subject that has received limited scientific attention. The intricate interplay of microbial communities, with a specific emphasis on lactic acid bacteria, within cocoa beans from the Navsari region, remains a largely unexplored domain.

This study endeavors to bridge this significant knowledge gap by conducting a comprehensive process of isolation, taxonomic classification, and detailed characterization of lactic acid bacteria sourced exclusively from cocoa beans in the Navsari region. Employing meticulous *in vitro* assessments, the objective of this research is to illuminate the distinct attributes and potential functionalities inherent to the LAB strains originating from this specific geographical area. The anticipated outcomes of this study bear the potential to furnish innovative insights into the cocoa fermentation process, potentially paving the way for the introduction of novel strategies aimed at augmenting the quality and safety of cocoa products. By shedding light on the unique microbial dynamics present in the Navsari cocoa beans, this research could contribute significantly to the broader understanding of cocoa fermentation processes and open avenues for advancing cocoa-related industries.

## 2. Materials and methods

### 2.1. Collection of samples

The investigation employed Forastero varieties of *Theobroma cacao L.* featuring mature yellow pods, which were procured from the Navsari Horticulture Centre situated in Navsari (20°55′38′′ N, 72°53′54′′ E), South Gujarat, India. The procedure for harvesting the fruits encompassed manual extraction, followed by a meticulous separation of seeds and their associated pulp from the placental tissue. Subsequently, both the seeds and pulp were fastidiously arranged within the sterilized fermentation container. In anticipation of the fermentation phase, precise 50-gram aliquots of seeds and their corresponding pulp were systematically sampled from diverse locations within the fermentation container. The process of sample collection adhered rigorously to aseptic principles, employing sterile polyethylene bags as the chosen containment method. For preservation during transit, the gathered samples were maintained under refrigeration at 5 ± 1°C until their arrival for testing at the laboratory.

### 2.2. Isolation of lactic acid bacteria from fermented cacao fruit

For the isolation process of LAB from 24 to 72 h of fermented cacao, a mixture of 50 grams of cacao seeds and pulp was prepared with 450 mL of water containing 0.1% peptone. The viable bacterial cell count was determined using the pour-plate technique. The rinse water underwent serial dilution using 0.1% peptone water. Subsequently, 100 μL of the diluted samples were inoculated onto deMan, Rogosa, and Sharpe (MRS) agar plates (Himedia, Mumbai, India). The plates were then incubated at 37°C for 24–48 h in anaerobic conditions. The visible colonies were counted, and the cell counts were converted to colony-forming units per milliliter (CFU/mL).

### 2.3. Confirmation and characterization tests of LAB isolates

#### 2.3.1. Potassium hydroxide (KOH) test

To ascertain the Gram reaction of LAB isolates, the potassium hydroxide (KOH) test was used. The LAB cultures were cultivated at 37°C for 24 h on MRS agar. To perform the test, a clean slide was prepared, and a drop of 3% aqueous KOH was placed on it. Using a sterile loop, visible cells from fresh cultures were transferred into the KOH drop. The mixture of cells and KOH was thoroughly mixed and frequently swirled over an area of approximately 1–2 cm^2^ on the slide. Based on the absence of a viscid product, the isolates that did not exhibit such a characteristic were selected, indicating their Gram-positive phenotype as lactic acid bacteria (Powers, 1995).

#### 2.3.2. Catalase test

The isolates were cultured on MRS agar and incubated at 37°C for 24 h to facilitate overnight growth. For the catalase test, a 24-h-old culture was placed on a glass slide, and two drops of 3% hydrogen peroxide were added. The presence of oxygen bubbles indicated a positive catalase reaction, suggesting the production of the catalase enzyme by the tested bacteria. Consequently, only isolates that did not demonstrate the formation of gas bubbles were chosen for further investigation.

#### 2.3.3. Spore staining

LAB isolates, identified as Gram-positive and catalase-negative, were cultivated on MRS agar at a temperature of 37°C for 24 h. The spore-staining method was employed to evaluate the presence of endospores. Under light microscopy using oil immersion objectives, the development of endospores was studied using the spore-staining method. Only the isolates that did not demonstrate endospore production were chosen for additional examination.

### 2.4. *In vitro* evaluation of probiotic characteristics

#### 2.4.1. Assessment of acid tolerance in lactic acid bacteria

The LAB (lactic acid bacteria) isolates were subjected to overnight incubation in MRS broth at 37°C to promote their active growth. Centrifugation was then used to separate the cells (7000 rpm, 4°C, 10 min). A control group with a pH of 6.5 was maintained using MRS broth, while an acidic environment was created by adjusting the pH of the MRS broth to pH 1.0, 2.0, and 3.0 by adding 1 N HCl (Ramos et al., 2013). Following harvest, the cells were maintained at 37°C in an incubator while suspended in MRS broth with an acidic pH. Samples were taken and serially diluted in phosphate-buffered saline (PBS) at set intervals of 0, 1, and 2 h, respectively. After plating these dilutions onto MRS agar plates, the isolates were incubated for 48 h at 37°C. The plate count technique was used to determine the vitality of the cells, and the findings were represented as log_10_ CFU/mL. This experimental technique enabled the examination of the ability of LAB isolates to tolerate and survive in an acidic environment, revealing valuable insights into their resilience under such circumstances.

#### 2.4.2. Assessment of bile tolerance in lactic acid bacteria

The bile tolerance of LAB (lactic acid bacteria) isolates was demonstrated according to the methodology outlined by Ramos et al. (2013). The LAB culture isolates were collected, and their pellets were obtained using the previously described procedure. Following that, the pellets were reconstituted in 5 mL of MRS broth supplemented with 0.3 and 1% (w/v) oxgall bile salt (Sigma Aldrich, India). A control sample was also prepared without the addition of bile salts. Subsequently, the samples were incubated at 37°C and collected at specific time intervals of 0, 2, and 3 h. To determine the viability counts, 100 μL samples were taken and plated onto MRS agar plates after performing appropriate serial dilutions. The number of viable cells was determined and expressed as log_10_ CFU/mL.

#### 2.4.3. Determination of bile salt hydrolase activity

Bile salt hydrolase (BSH) activity was evaluated according to the procedure outlined by Ayyash et al. (2018). This method involved the measurement of amino acids released from conjugated bile salts, including 6.0 mmol/L sodium glycocholate, 6.0 mmol/L sodium taurocholate, and a mixture of conjugated bile salts consisting of 6.0 mmol/L sodium glycocholate and sodium taurocholate. The quantification of amino acids was achieved using ninhydrin, and the absorbance was measured at 570 nm using a spectrophotometer. One unit of BSH activity, measured in U/ml, denoted the enzyme quantity that could release 1 millimole of amino acid from the substrate within a minute. The evaluation of protein concentration utilized the Lowry method (Lowry et al., 1951), wherein bovine serum albumin (BSA) was used as the reference standard. Ensuring credibility, all experiments were conducted independently in triplicate.

#### 2.4.4. Determination of resistance to phenol

Probiotics must be phenol-resistant to survive in the gastrointestinal system, considering aromatic amino acids from dietary proteins can be deaminated to produce phenols. These phenolic compounds have the potential to inhibit the growth of LAB (Kılıç et al., 2013). In this study, overnight cultures of the probiotics were prepared and subsequently inoculated into MRS broth supplemented with 0.4% (w/v) phenol. The cultures were subjected to serial dilution and plated onto MRS agar plates at predetermined time intervals of 0 and 24 h. The viability of cells was determined using the plate count method, which enabled the quantification of viable cell numbers in log CFU/mL.

#### 2.4.5. *In vitro* survivability of LAB isolates in simulated gastric and pancreatic fluid

For assessment of the survivability of the isolated samples in simulated *in vitro* conditions mimicking gastric juice, the following procedure was carried out:

(1) Simulated juices preparation: the preparation of gastric and pancreatic juices followed the method outlined by Bhushan et al. (2021). (2) Harvesting and washing of overnight-grown LAB isolates: the isolated samples, which had been grown overnight for 24 h, were collected by centrifugation at 6000 rpm, 4°C, for 10 min. The resulting cell pellets were washed with phosphate-buffered saline (PBS) and then resuspended in gastric juice. The cells that were resuspended were adjusted to a concentration such that the final absorbance at 500 nm reached 1.2. (3) Simulation of peristaltic movement: the culture samples were exposed to a simulated movement that is similar to peristalsis, aiming to mimic the conditions found in the gastrointestinal tract. This was accomplished by placing the samples on a shaker operating at 200 rpm and maintaining a temperature of 37°C for 3 h. (4) Preparation of Serial dilutions and plating: serial dilutions were prepared, and aliquots of the culture samples were plated on MRS agar at two specific time points: 0 h (immediately after the addition of gastric fluid) and 3 h. This process enabled the quantification of viable bacterial cells by facilitating their enumeration. (5) Percentage survivability calculation: to determine the percentage survivability in the simulated gastric fluid, the following formula was employed:


$$\begin{array}{l} \mathrm{Survivability}\;\mathrm{in}\;\mathrm{gastric}\;\mathrm{fluid}\;\left(\%\right)\\ \;=\frac{\mathrm{Number}\;\mathrm{of}\;\mathrm{viable}\;\mathrm{cells}\;\mathrm{after}\;3\;\mathrm{hours}}{\mathrm{Number}\;\mathrm{of}\;\mathrm{viable}\;\mathrm{cells}\;\mathrm{at}\;0\;\mathrm{hours}}\;x\;100 \end{array}$$

To evaluate the survivability of the culture samples in simulated pancreatic juice conditions, the following steps were performed:

(1) Centrifugation and washing: after the 3-h incubation with gastric fluid, the culture was centrifuged at 6000 rpm for 12 min at 4°C. The resulting cell pellets were washed with phosphate-buffered saline at pH 7.3 to remove any residual gastric fluid. (2) Mixing with pancreatic fluid: the washed cell pellets were mixed with an equal volume of pancreatic fluid. (3) The culture samples, combined with pancreatic fluid, were subjected to incubation in an orbital shaker set at 200 rpm to replicate physiological gut conditions. The incubation was performed for 24 h at a temperature of 37°C. (4) Serial dilutions were prepared from the culture samples at two specific time points: 0 h (immediately after the addition of pancreatic fluid) and 24 h. The diluted samples were then spread on MRS agar plates after being diluted to a desired concentration. By employing this method, viable bacterial cells surviving under the simulated conditions of pancreatic fluid were quantified. (5) Calculation of percentage survivability: the percentage survivability calculation: to determine the percentage survivability in the pancreatic fluid, the following formula was employed:


$$\begin{array}{l} Pe\mathrm{rcentage}\;\mathrm{Survivability}\\ \;=\frac{\mathrm{Number}\;\mathrm{of}\;\mathrm{viable}\;\mathrm{cells}\;\mathrm{after}\;24\;\mathrm{hours}}{\mathrm{Number}\;\mathrm{of}\;\mathrm{viable}\;\mathrm{cells}\;\mathrm{at}\;0\;\mathrm{hours}}\;x\;100 \end{array}$$

#### 2.4.6. The assay of bacterial resistance to lysozyme

To assess the ability of selected LAB cultures to withstand the effects of lysozyme, a modified version of the method described by Zago et al. (2011), was employed. Initially, the LAB cultures were grown overnight in MRS broth and subsequently subjected to centrifugation to separate the cells from the growth medium. The resulting cell pellets were washed twice with PBS solution (pH 7.0) to get rid of any residual media components. A sterile electrolyte solution comprising NaCl (6.2 g/L), CaCl_2_ (0.22 g/L), NaHCO_3_ (1.2 g/L), and KCl (2.2 g/L) was created to approximate salivary conditions seen *in vivo*. The solution was fortified with 100 mg/L of lysozyme enzyme (HiMedia, Mumbai, India). A 10-μL suspension of LAB cells was then added to the sterile electrolyte solution.

#### 2.4.7. Evaluation of cell auto-aggregation

The capacity of bacteria to engage in auto-aggregation plays a pivotal role in the persistence of their population within the gastrointestinal environment as proposed by Rickard et al. (2003). Cell auto-aggregation was evaluated using a modified version of the method described by Juárez Tomás et al. (2005) with some minor modifications. The bacterial cultures were grown in MRS broth for approximately 16–18 h to facilitate growth and establishment. Subsequently, the cells were harvested by centrifugation, followed by washing, and resuspended in PBS (phosphate-buffered saline) at pH 7.0. The suspension was subsequently adjusted to achieve a predetermined cell density, which was determined by measuring the absorbance at 600 nm and aiming for a value of 0.5. This standardized suspension was then incubated for 2 h at 37°C to facilitate the auto-aggregation process. Following the incubation period, a volume of one milliliter from the upper phase of the suspension was extracted with caution, and its absorbance at 600 nm was measured with the help of a spectrophotometer. Cell auto-aggregation was assessed by quantifying the reduction in absorbance compared to the initial absorbance measured at 600 nm. The following equation was utilized for calculating cell auto-aggregation:


$$\mathrm{Auto}-\mathrm{aggregation}\;\left(\%\right)=\left[\frac{\left(Xo-X1\right)}{Xo}\right]x\;100$$

Here, X_0_ indicates the absorbance at 0 h, and X_1_ indicates the absorbance after 2 h of incubation at 37°C.

#### 2.4.8. Evaluation of cell co-aggregation

The investigation involved generating a cell suspension by combining pathogenic strains at a 2:1 ratio, encompassing *Bacillus cereus* MTCC 430, *Escherichia coli* MTCC 443, and *Pseudomonas aeruginosa* MTCC 424. This composite solution was then subjected to a 37°C incubation for 2 h. The resultant culture was assessed for its light absorbance at a wavelength of 600 nm, and subsequent analysis was conducted following a methodology akin to the procedure outlined by Tatsaporn and Kornkanok (2020).

To gauge the extent of coaggregation, the coaggregation percentage was calculated using the subsequent equation:


$$Co-\mathrm{aggregation}\;\left(\%\right)=\left[\frac{\left(X1-X0\right)}{X1}\right]x\;100$$

Where X0 = Absorbance of LAB and Pathogen mixture at 4 h, X1 = Initial Absorbance of LAB and Pathogen mixture at 0 h.

#### 2.4.9. Evaluation of cell surface hydrophobicity

A modified version of the technique reported by Ahire et al. (2021), was employed to scientifically assess the adherence of LAB isolates to the hydrocarbon solvents xylene and chloroform. First, a cell culture that flourished overnight was separated by centrifuging it for 10 min at 8000 rpm at 4°C. After being rinsed twice with PBS (pH 7.0), the cell pellets were suspended in a sterile solution of 0.1 M KNO_3_ (pH 6.2). The initial optical density (OD) of the cell suspension was carefully adjusted to fall within the range of 0.55–0.66 when measured at 600 nm. Then, 3 mL of the cell suspension and 1 mL of the hydrocarbon solvent xylene were combined in a 3:1 ratio. The mixture was vortexed for 1 min after being incubated at 37°C for 10 min to facilitate the way the bacterial cells and hydrocarbon solvent interact. The aqueous phase (1 mL) was cautiously withdrawn following a 30-min incubation period at 37°C. At 600 nm. The final optical density (OD) of the aqueous phase (X1) was determined. The hydrophobicity percentage was determined by calculating the reduction in absorbance using the following formula:


$$\mathrm{Cell}\;\mathrm{surface}\;\mathrm{hydrophobicity}\;\left(\%\right)=\left[1-\frac{X1}{Xo}\right]x\;100$$

The percentage of hydrophobicity was calculated by comparing the initial optical density (OD), denoted as X0, of the suspended culture before incubation with the final optical density (OD) of the aqueous phase after incubation, represented as X1.

The assessment of cell-surface hydrophobicity offers valuable insights into the adhesive capabilities of LAB isolates to hydrocarbon surfaces, which implies their potential for attachment to gut epithelial cells.

#### 2.4.10. Evaluation of exopolysaccharide production

Exopolysaccharide synthesis was investigated by streaking cultures that were grown overnight onto the surface of plates containing a medium composed of ammoniated ruthenium oxychloride (ruthenium red) dye-incorporated milk. This medium was formulated with 2% w/v sucrose, 10% w/v skim milk powder, and 0.08 g/L ammoniated ruthenium oxychloride, solidified using 1.5% w/v agar (Angmo et al., 2016).

The quantitative evaluation of exopolysaccharide (EPS) production was conducted using a standardized protocol, following the approach described by Abushelaibi et al. (2017). The assessment of EPS production from the five selected LAB strains took place within MRS broth supplemented with 2% sucrose. Cultures of these LAB strains, cultivated overnight in MRS, were subjected to boiling at 100°C for 30 min. Upon cooling, the cultures underwent treatment with a 4% (v/v) solution of 85% trichloroacetic acid and were subsequently centrifuged at 4°C with a speed of 12000 rpm for 30 min. This centrifugation step facilitated the separation of cellular materials and proteins.

Following centrifugation, the supernatant was mixed with ethanol in a 4:1 ratio, resulting in the pellet’s resuspension. This mixture was then kept at 4°C to promote the precipitation of crude EPS. The crude EPS was recovered through another round of centrifugation at 4°C, utilizing a speed of 12000 rpm for 30 min. The resulting pellet was dissolved in 1 mL of double-distilled water. The total EPS content in each sample was quantified through the phenol–sulfuric acid method, utilizing glucose as a standard (ranging from 5 to 100 mg/L). The carbohydrate assay underwent calibration using a mixture composed of d-galactose, d-glucose, and l-rhamnose in a proportional ratio of 5:1:1, as documented by Gruter et al. (1993). The reported results are expressed in mg/mL of carbohydrate. Regarding the EPS measurements, the presented values were determined by subtracting the background interference amount found in the non-inoculated medium (approximately 50 mg of carbohydrate per liter) from the quantity observed in the fermented broth (Kimmel et al., 1998).

#### 2.4.11. α-Glucosidase inhibitory activity of LAB isolates

The assessment of α-glucosidase inhibitory activity was carried out with slight modifications following the procedure detailed by Reuben et al. (2019). For this purpose, α-glucosidase derived from yeast with an activity of 100 U/mg was employed. The test samples of cell-free supernatant (CS), cell-free extracts (CE), and intact cells (IC) – 100 μL each were mixed with 50 mM PBS buffer at pH 6.8 and allowed to incubate for 10 min. Subsequently, α-glucosidase enzyme (0.25 U/mL, 100 μL) was added and pre-incubated for 15 min at 37°C. The addition of 5 mM pNPG (p-nitrophenol-D-glucopyranoside, 100 μL) was followed by further incubation for 30 min at 37°C. The enzymatic reaction was quenched using 1000 μL of 0.1 M Na_2_CO_3_, and the absorbance of 4-nitrophenol was measured at 405 nm. The percentage of inhibition was calculated as per the following equation:


$$\alpha \;\mathrm{glucosidase}\;\mathrm{inhibition}\;\left(\%\right)=\left[1-\frac{X1}{Xo}\right]x\;100$$

Where ‘X2’ represents the absorbance of the reactants without the sample, and ‘X1’ represents the absorbance of the reactants combined with the sample.

### 2.5. Antioxidant performance of LAB

#### 2.5.1. Radical scavenging rate determination by DPPH assay

The procedure outlined by Elfahri et al. (2014) was adopted to execute this analysis. The evaluation of cellular radical scavenging efficacy was conducted using the DPPH (1,1-diphenyl-2-picrylhydrazyl) assay at cell concentrations of 10^3^, 10^6^, and 10^9^ CFU/mL. The radical scavenging activity was quantified using the subsequent formula.


$$\mathrm{DPPH}\;\mathrm{Scavenging}\;\mathrm{rate}\;\left(\%\right)=\left[1-\frac{X1}{Xo}\right]x\;100$$

Absorbance with Sample (X1) signifies the absorbance of the reaction involving the sample.

Absorbance without Sample (X0) represents the absorbance of the reaction in the absence of the sample.

#### 2.5.2. Radical scavenging rate determination by ABTS assay

The approach outlined by Soleymanzadeh et al. (2016) was employed to evaluate the radical scavenging rate exhibited by cells at concentrations of 10^3^, 10^6^, and 10^9^ CFU/mL using the 2,2′-azino-bis 3-ethylbenzothiazoline-6-sulfonic acid (ABTS) assay. The radical scavenging activity was quantified using the subsequent formula.


$$\mathrm{ABTS}\;\mathrm{Scavenging}\;\mathrm{rate}\;\left(\%\right)=\left[1-\frac{X1}{Xo}\right]x\;100$$

### 2.6. Assessment of safety aspects

#### 2.6.1. Assessment of DNase activity

A modified version of the technique described by Sangprapai et al. (2022), was employed to measure the deoxyribonuclease activity of the isolates. The LAB cultures were grown in MRS broth at 37°C for 18–24 h and adjusted to a concentration of 10^8^ CFU/mL. To assess the DNase activity, the LAB cultures were streaked onto DNase test agar plates and subsequently incubated at a temperature of 37°C for 24 h. Following the incubation period, the plates were treated with 1 N HCl, and the presence of DNase activity was indicated by clear zones surrounding the bacterial streaks.

#### 2.6.2. Assessment of hemolytic activity

The hemolytic assay was conducted to assess the hemolytic activity of the isolated LAB strains. This was achieved using blood agar (HiMedia, Mumbai, India) as described by Halder et al. (2017). The evaluation entailed observing the degree of red blood cell lysis surrounding the bacterial colonies. A key indicator of hemolytic reaction was the presence of a clear zone around the colony, signifying the extent of hemolytic activity displayed by the isolated strains.

#### 2.6.3. Assessment of antibiotic susceptibility

The susceptibility of LAB isolates to antibiotics was evaluated using the antibiotic disc diffusion technique, as described in the study conducted by Singh et al. (2012). Soft MRS agar plates with a concentration of 0.7% w/v were prepared by pouring the medium into plates and allowing it to solidify at room temperature. The MRS plates were then uniformly inoculated with 100 μL of freshly grown bacterial cultures, and the agar plates were left undisturbed to air-dry. Antibiotic discs containing specific antibiotics were carefully placed on the inoculated plates. Subsequently, the plates were incubated for at least 48 h at 37°C to facilitate bacterial growth and the formation of inhibition zones around the antibiotics. Following the incubation period, the diameter of the inhibition zones was measured using a zone-diameter measuring device. The results obtained were analyzed and interpreted based on the interpretative zone diameters provided by the Performance Standards for Antimicrobial Disc Susceptibility Tests (CLSI, 2007), categorizing the isolates as susceptible, intermediate, or resistant. The following antibiotics were utilized in the assessment of antibiotic susceptibility: Vancomycin (30 μg), Penicillin G (10 μg), Amoxicillin (10 μg), Kanamycin (30 μg), Tetracycline (30 μg), Chloramphenicol (30 μg), Streptomycin (10 μg), and Gentamicin (10 μg).

#### 2.6.4. Assessment of antimicrobial activity

The antimicrobial activity of the isolates against pathogenic strains was assessed using the agar-well diffusion method, following the protocol described by Ridwan et al. (2008). The pathogenic strains used in the study included *Escherichia coli* MTCC 443, *Klebsiella pneumoniae* MTCC 3384, *Pseudomonas aeruginosa* MTCC 424, *Bacillus cereus* MTCC 430, and *Staphylococcus aureus* MTCC 737. To conduct the assay, 100 μL of each pathogen was mixed with soft agar and spread onto Muller Hinton Agar (MHA) plates. Wells was created on the plates using a borer. Then, 100 μL of an overnight culture of LAB was added to each well. The plates were left to dry and then incubated at 37°C for 24 to 48 h. After incubation, the plates were examined for the presence of a zone of inhibition (ZOI) around each well. The size of the ZOI indicated the antimicrobial activity of the LAB isolates against the tested pathogens.

### 2.7. Characterization of isolates based on physiological and biochemical tests

The isolates were identified using morphological and biochemical techniques. Gram staining methods were employed for morphological identification, enabling microscopic examination of the isolates. Furthermore, tests for catalase, oxidase, motility, and endospores were carried out to identify the particular traits of isolates. Only non-motile, non-spore-forming, catalase-negative, and Gram-positive isolates were subjected to further analysis for identification. To discover more about the isolates, biochemical assays were carried out, including the IMViC tests as suggested in Bergey’s Manual of Determinative Bacteriology. Moreover, the capacity of LAB isolates to ferment various carbohydrates was evaluated. Based on their distinctive physiological and biochemical characteristics, these approaches are intended to characterize and distinguish the isolates.

### 2.8. Molecular identification of the potential probiotic isolate

Isolates were chosen based on their probiotic attributes, and their genus was identified by using suitable primers designed to amplify a 250-bp region of the 16S rRNA gene. Genotypic identification of the isolates was conducted by performing PCR with these genus-specific primers. The PCR reaction mixture (25 μL) included dNTPs (0.2 mM), DNA template (0.5 μL), forward and reverse primers (10 pmol each), Taq polymerase (0.5 U), and 1x PCR buffer. The optimal PCR conditions were as follows: an initial denaturation step at 95°C for 5 min, followed by 35 cycles of denaturation at 95°C for 1 min, annealing at 55°C for 1 min, and extension at 72°C for 5 min. A final extension step was performed at 72°C for 7 min. The PCR products were separated on a 0.8% agarose gel in 1x TAE buffer (pH 8) containing ethidium bromide (2 μL). A total of 5 μL of the PCR product mixed with loading dye was loaded onto the gel. The best isolate, selected based on probiotic properties among the six isolates, was further identified by sequencing the 16S rRNA gene. Universal primers 357f (5′-*CTCCTACGGGAGGCAGCAG*-3′) and 1391r (5′-*GACGGGCGGTGTGTRCA*-3′) were used for the sequencing reaction. The PCR product was sent for commercial sequencing, and the resulting sequence was analyzed and compared using the BLASTn tool for identification purposes. The phylogenetic tree was constructed by the neighbor-joining method using MEGA 11.0 software.

### 2.9. Statistical analysis

The experiments were conducted in triplicate to ensure reproducibility. Mean values and their respective standard deviations were calculated for each experiment. The statistical analysis of the data was performed using SPSS software (Version 29.0.1.0, SPSS, Chicago, IL, United States). To assess the differences in means among different treatment groups, a one-way ANOVA followed by Duncan’s multiple range test was employed, with a significance threshold set at *p* < 0.05.

## 3. Results

### 3.1. Primary characterization of LAB isolated from *Theobroma cacao L.*

A total of 11 isolates were isolated from the *Theobroma cacao L.* Forastero variety under investigation in this study. Among these, five isolates exhibited phenotypic traits characteristic of LAB. Specifically, two isolates displayed a cocci morphology, while three isolates exhibited rod-like morphology. These isolates were Gram-positive, anaerobic, catalase-negative, oxidase-negative, non-spore-forming, and non-motile. Biochemical profiling indicated their hetero-fermentative nature, with no production of gas from glucose fermentation. Under optimal conditions at 37°C, all isolates demonstrated conventional growth, with strains CYF2, CYF3, and CR2 displaying resilience at temperatures up to 45°C. In terms of carbohydrate utilization, all five isolates were capable of fermenting glucose, fructose, ribose, galactose, mannitol, xylose, maltose, sucrose, and lactose (Supplementary Table 1).

### 3.2. Determination of acid and bile tolerance in LAB

**Figure 1:**
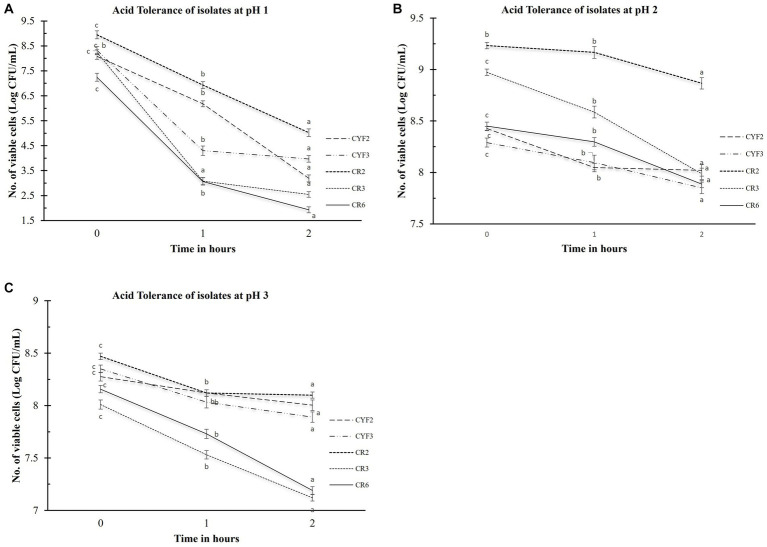
Acid tolerance of five distinct LAB isolates, measured as Log CFU/mL. The experiment involves different pH levels- pH1 **(A)**, pH2 **(B)**, and pH3 **(C)**, with 1-h and 2-h incubations at 37°C. The values depict the mean ± SD of three replicate assays. Statistical analysis (One-way ANOVA and Duncan’s multiple range tests) identifies significant differences (*p* ≤ 0.05) in survival rates within the 1-h interval marked by superscripts (a–c).

The evaluation of acid tolerance was systematically carried out within the pH conditions of 1, 2, and 3. This analysis holds significance as the ability of probiotic strains to withstand the acidic environment of the stomach profoundly impacts their efficacy. The survivability of these bacterial strains after a 2-h exposure to these acidic conditions was quantified by assessing viable cell counts, expressed in Log CFU/mL. The outcomes, as portrayed in Figure 1, elucidate the extent of this survivability. The logarithmic reduction in CFU/mL for pH1 ranged from 3.92 for CR2 to 5.69 for CR3. Within the pH2 environment, the range spanned from 0.36 CFU/mL for CR2 to 0.98 CFU/mL for CR3. As for the pH3 condition, the values fluctuated between 0.27 CFU/mL for CYF2 and 0.96 CFU/mL for CR6. Isolate CR2 and CYF3 displayed significant survival in acidic conditions. Whereas, isolate CR3 and CR6 displayed sensitivity to acidic environment.

**Figure 2:**
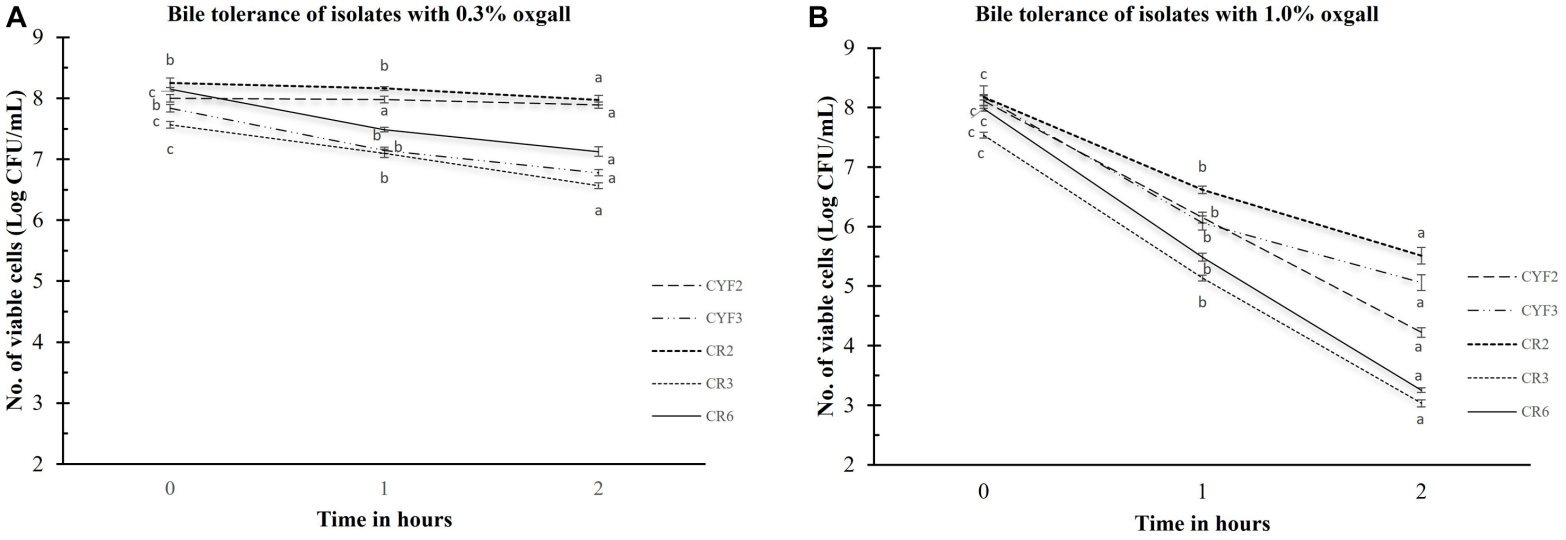
Bile tolerance of five distinct LAB isolates, measured as Log CFU/mL. The experiment involves different bile salt concentrations 0.3% oxgall **(A)**, and 1.0% oxgall **(B)**, with 2-h and 3-h incubations at 37°C. The values depict the mean ± SD of three replicate assays. Statistical analysis (One-way ANOVA and Duncan’s multiple range tests) identifies significant differences (*p* ≤ 0.05) in survival rates within the time interval marked by superscripts (a–c).

Bile salt tolerance plays a pivotal role in governing bacterial metabolic functions and their potential for establishing colonization within the small intestine. In the pursuit of comprehending this aspect, the quantification of viable cell counts in Log CFU/mL for the isolated strains was meticulously conducted. This assessment followed a rigorous 3-h exposure to oxgall bile salts at varying concentrations of 0.3 and 1.0% (w/v), as graphically depicted in Figure 2. After the 3-h interaction with the bile salts, the examined strains conspicuously maintained substantial levels of viable cells. In terms of logarithmic reduction, within the 0.3% oxgall bile salt concentration, the spectrum extended from 0.28 for CR2 to 1.38 for CYF3. Correspondingly, at the higher 1.0% oxgall bile salt concentration, the values ranged from 2.66 for CR2 to 4.73 for CR6. Isolate CR2 displayed significant survival in bile salt while CR6 displayed greater susceptibility to bile salt.

### 3.3. Determination of bile salt hydrolase activity

The outcomes of the Bile Salt Hydrolase (BSH) assessments for the five isolates are comprehensively presented in Table 1. The specific activity of BSH was observed within a range of 0.42–2.23 U/mg for sodium glycocholate, 0.30–2.86 U/mg for sodium taurocholate, and 0.34–3.55 U/mg for the bile salts mixture. Isolates CR2 and CYF3 notably exhibited elevated BSH activities against all three tested bile salts.

**Table 1:** Bile salt hydrolase activity in U/mL of five distinct LAB isolates.

Isolates	Sodium glycocholate	Sodium taurocholate	Bile salts mixture
CYF2	0.76 ± 0.20^bc^	0.81 ± 0.05^bc^	0.76 ± 0.20^b^
CYF3	2.23 ± 0.26^ab^	2.32 ± 0.70^ab^	3.55 ± 0.15^a^
CR2	3.03 ± 0.52^a^	2.86 ± 0.21^a^	2.79 ± 0.27^a^
CR3	0.42 ± 0.08^c^	0.30 ± 0.02^c^	0.42 ± 0.08^b^
CR6	0.56 ± 0.10^bc^	0.74 ± 0.43^bc^	0.34 ± 0.05^b^

The values depict the mean ± SD of three replicate assays. Statistical analysis (One-way ANOVA and Duncan’s multiple range tests) identifies significant differences (*p* ≤ 0.05) marked by superscripts (a–c).

### 3.4. Determination of resistance to phenol

**Figure 3:**
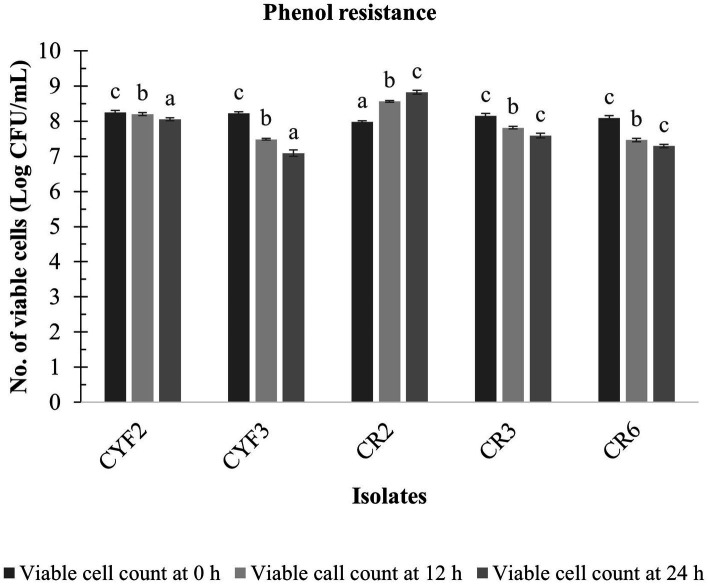
Phenol resistance of five distinct LAB isolates, measured as Log CFU/mL at 12 and 24 h incubation at 37°C. The values depict the mean ± SD of three replicate assays. Statistical analysis (One-way ANOVA and Duncan’s multiple range tests) identifies significant differences (*p* ≤ 0.05) in survival rates within the time interval marked by superscripts (a–c).

The LAB isolates that exhibited resilience in the presence of gastric fluid underwent an assessment of phenol resistance, revealing diverse levels of susceptibility to phenol (0.4% w/v). Notably, among these isolates, CR2 showcased pronounced survival capabilities and diminished sensitivity to phenol, leading to a substantial augmentation in viable cell counts throughout the incubation period. The cell viability increased from 7.98 to 8.82 log CFU/mL. However, isolates CYF3 and CR6 displayed greater susceptibility to phenol in comparison to the other isolates, as illustrated in Figure 3.

### 3.5. *In vitro* survivability of isolates in simulated gastric fluid and pancreatic fluid

**Figure 4:**
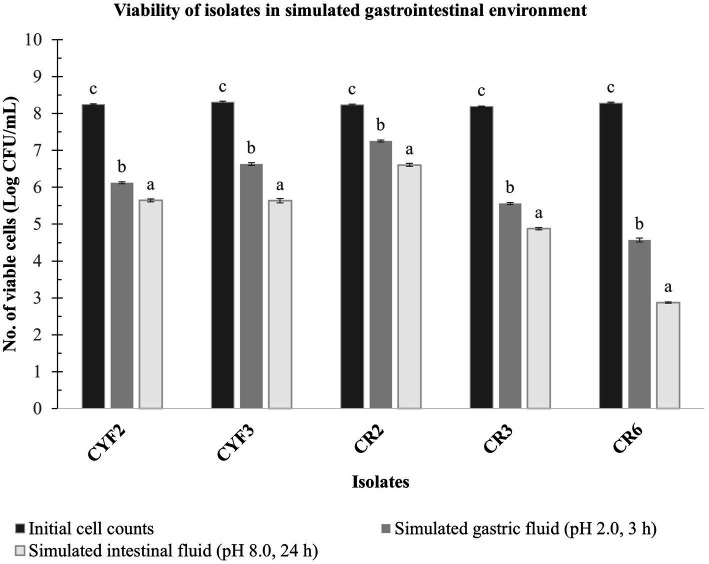
*In vitro* resistance to simulated gastric fluid pH-2.0, 3 h incubation and simulated intestinal fluid pH-8.0, 24 h incubation at 37°C. The values depict the mean of Log CFU/mL ± SD of three replicate assays. Statistical analysis (One-way ANOVA and Duncan’s multiple range tests) identifies significant differences (*p* ≤ 0.05) in survival rates within the time interval marked by superscripts (a–c).

A laboratory experiment was conducted to assess the combined impact of gastric and pancreatic fluids on the LAB isolates. Impressively, all isolates demonstrated viability following exposure to these fluids, highlighting their inherent ability to endure these conditions and potentially reach the intestinal environment. The viable cell count of the LAB isolates was quantified, and the outcomes are illustrated in Figure 4.

Isolate CR2 exhibited a slight reduction in the logarithmic count after a 3-h exposure to simulated gastric fluid and a 24-h exposure to simulated pancreatic fluid. Specifically, it reached a maximum logarithmic count of 7.25 CFU/mL after gastric fluid exposure and 6.60 CFU/mL after pancreatic fluid exposure. Similarly, isolate CYF3 displayed logarithmic counts of 6.63 CFU/mL and 5.63 CFU/mL for gastric and pancreatic fluid exposures, respectively. Conversely, the CR6 isolate displayed the lowest logarithmic counts, recording 4.57 and 2.87 CFU/mL for gastric and pancreatic fluid exposures, respectively.

### 3.6. Evaluation of lysozyme resistance of LAB

**Figure 5:**
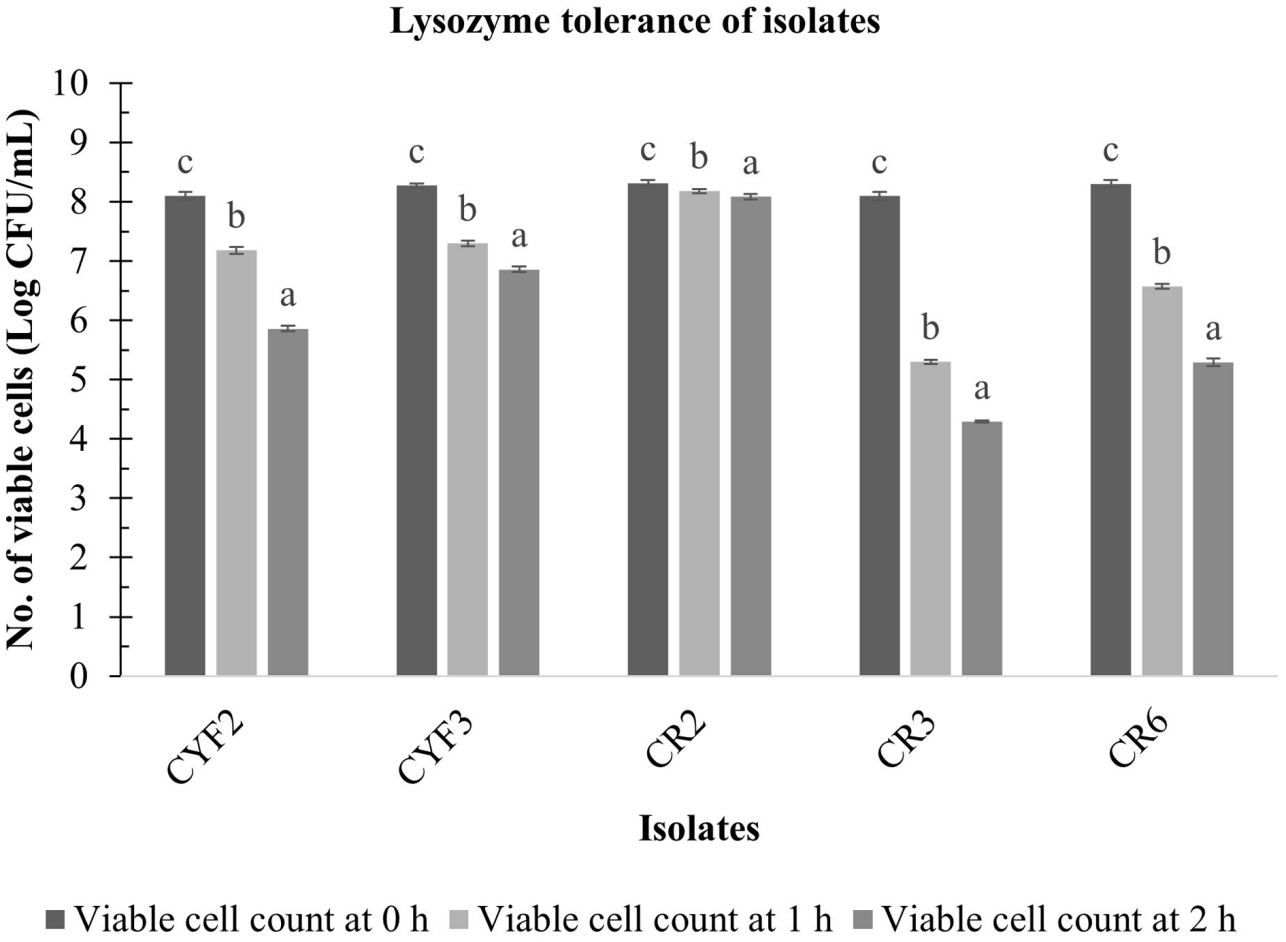
Lysozyme tolerance of five distinct LAB isolates, measured as Log CFU/mL at 0-h, 1-h, and 2-h incubation at 37°C. The values depict the mean ± SD of three replicate assays. Statistical analysis (One-way ANOVA and Duncan’s multiple range tests) identifies significant differences (*p* ≤ 0.05) in survival rates within the time interval marked by superscripts (a–c).

The resistance of five LAB isolates to lysozyme over a 60-min incubation at 37°C is depicted in Figure 5. Notably, the growth of these isolates exhibited a significant reduction (*p* < 0.05) as the incubation time progressed in the presence of 100 mg/L of lysozyme. The analysis of variance (ANOVA) confirmed the influence of incubation time on the resistance of isolates to lysozyme. Figure 5 further reveals that the decline in LAB growth in the presence of lysozyme ranged from 0.23 to 3.80 logarithmic units after the 60-min incubation period. Among the isolates, CR2, CYF3, and CYF1 demonstrated the highest resistance, while isolates CR3 and CR6 exhibited greater resilience.

### 3.7. Cell surface properties of LAB

**Figure 6:**
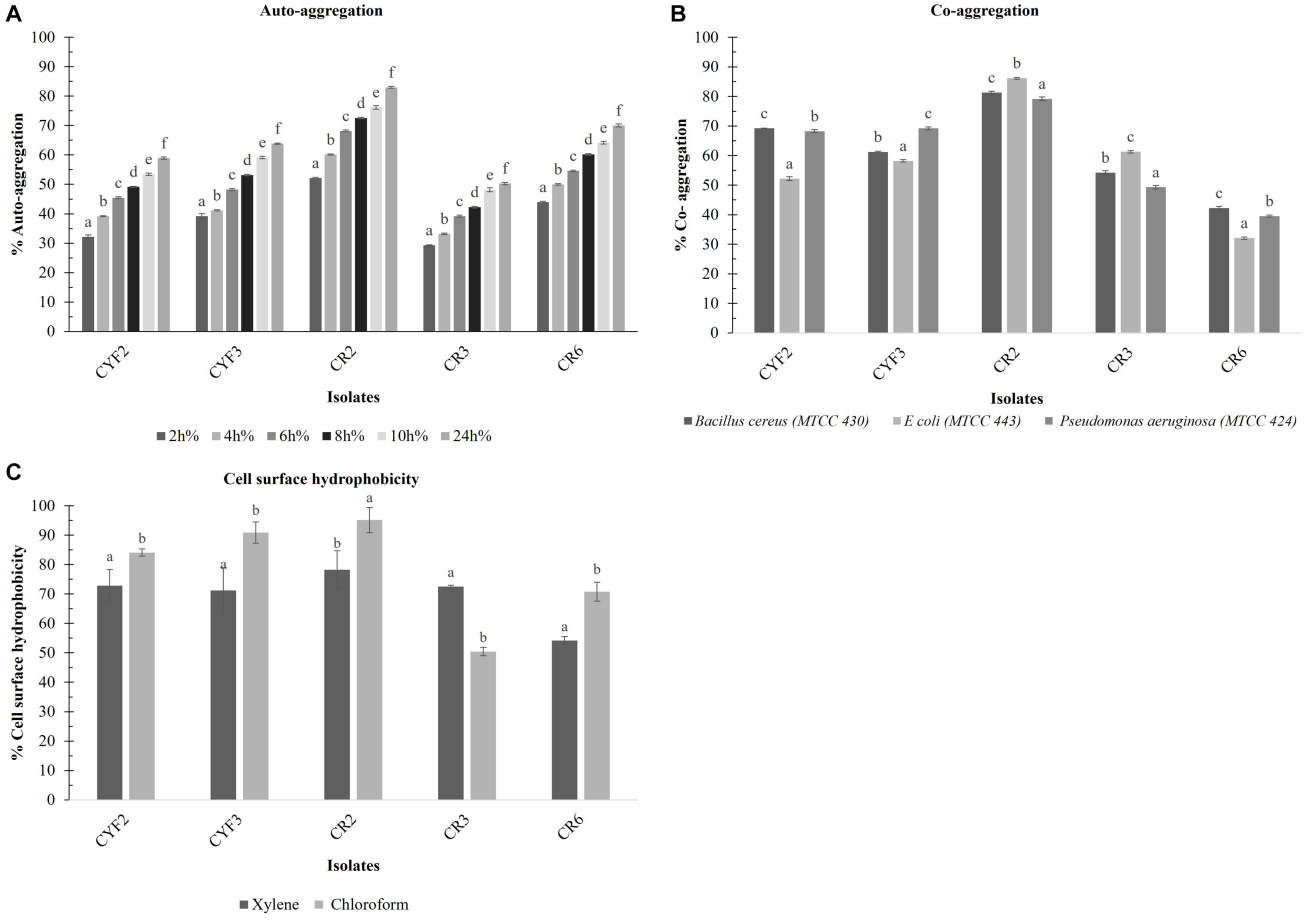
Cell surface properties of five distinct LAB – % Auto- aggregation at the interval of 2 h incubation at 37°C **(A)**, % Co- aggregation with *B. cereus*, *E. coli,* and *Ps. aeruginosa* **(B)**, and % Cell surface hydrophobicity with xylene and chloroform **(C)**. The values depict the mean ± SD of three replicate assays. Statistical analysis (One-way ANOVA and Duncan’s multiple range tests) identifies significant differences (*p* ≤ 0.05) marked by superscripts (a–f).

A consistent rise in the auto-aggregation percentage was evident across all isolates between the 2 and 24-h time points. The auto-aggregation activity of all isolates spanned a range from 53.29 to 81.96%. Notably, Isolate CR2 exhibited the highest auto-aggregation percentage, reaching 82.97% at the 24-h mark. Whereas CR3 displayed a relatively lower auto-aggregation percentage of 50.29% as displayed in Figure 6A.

The outcomes of co-aggregation involving LAB isolates and target pathogens are visually depicted in Figure 6B. Among these interactions, the most notable co-aggregation rates with *Bacillus cereus*, *Escherichia coli*, and *Pseudomonas aeruginosa* were observed with isolate CR2, showing percentages of 81.3, 86.15, and 79.17%, respectively. In contrast, the least co-aggregation with all three pathogens was observed with CR6, displaying rates of 42.21, 32.09, and 39.50%, respectively.

The isolates underwent evaluation for their cell surface hydrophobicity, a metric utilized to estimate their adhesive potential, employing xylene and chloroform as indicators are displayed in Figure 6C. Notably, a statistically significant difference (*p* < 0.05) in hydrophobicity was discerned among the diverse test strains. In terms of xylene-based cell surface hydrophobicity, the range extended from a minimum of 54.13% for CR6 to a maximum of 79.82% for CR2. Conversely, when chloroform was employed, the range of cell surface hydrophobicity spanned from a minimum of 50.45% for CR3 to a maximum of 95.12% for CR2.

### 3.8. Exopolysaccharide production of LAB isolates

**Figure 7:**
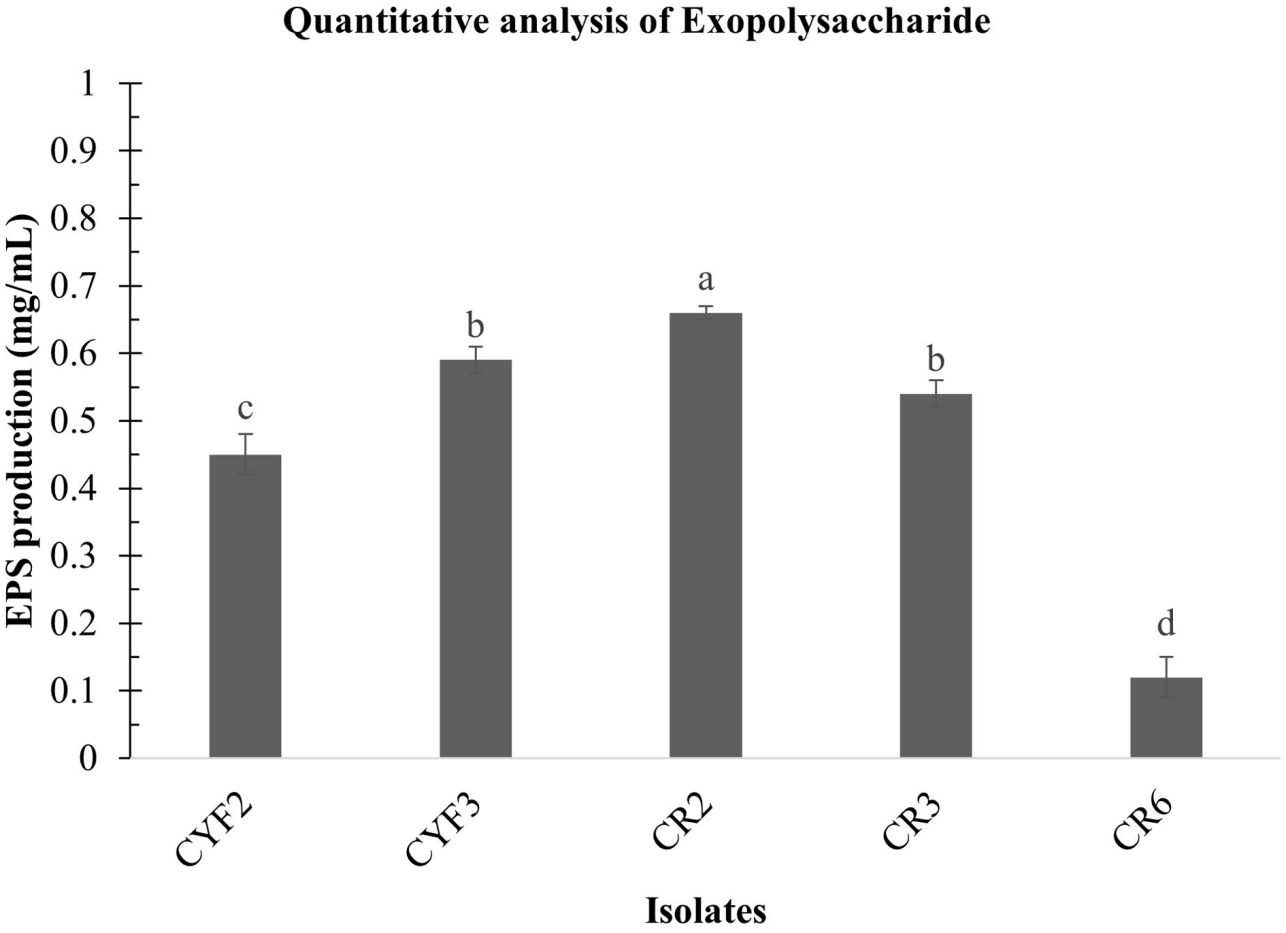
Quantitative analysis of exopolysaccharide from five distinct LAB isolates measured as mg/mL of EPS. The values depict the mean ± SD of three replicate assays. Statistical analysis (One-way ANOVA and Duncan’s multiple range tests) identifies significant differences (*p* ≤ 0.05) marked by superscripts (a–d).

In the present research, LAB obtained from *Theobroma cacao L.* was subjected to a comprehensive analysis of both qualitative and quantitative aspects of Exopolysaccharide (EPS) production. The agar medium supplemented with ammoniated ruthenium oxychloride (ruthenium red) milk facilitated the growth of distinctive white, viscous colonies of the LAB isolates. Among these, CR2 demonstrated the highest EPS production, quantified at 0.66 mg/mL. Following closely, CYF3 exhibited an EPS production of 0.59 mg/mL, while CR3 and CYF2 yielded productions of 0.54 mg/mL and 0.45 mg/mL, respectively. Notably, the strain CR6 displayed the lowest EPS production, measuring only 0.12 mg/mL of exopolysaccharides as described in Figure 7.

### 3.9. α-Glucosidase activity of LAB isolates

The assessment of inhibitory effects on the α-glucosidase enzyme involved the utilization of CS, CE, and IC derived from the five isolates. These constituents were employed to gauge their impact on the enzymatic activity of α-glucosidase. Across all isolates, both CS and CE demonstrated significant modulatory effects on α-glucosidase activity. The range of α-glucosidase inhibition achieved by employing CS, CE, and IC spanned from 12.08 to 56.55%. Notably, strain CR2 exhibited the highest α-glucosidase inhibition rate at 56.55%. Comparative analysis of the three constituents (CS, CE, and IC) revealed that intact cells exhibited the lowest degree of inhibition compared to the supernatant and pellets as presented in Table 2.

**Table 2:** *α*-Glucosidase inhibition activity of five distinct LAB isolates in percentage.

Isolates	*α*-glucosidase inhibition (%)		
	CS	IC	CE
CYF2	42.40 ± 2.45^a^	12.08 ± 3.20^a^	39.12 ± 2.14^a^
CYF3	54.26 ± 4.24^c^	22.57 ± 4.21^c^	48.42 ± 4.98^c^
CR2	56.55 ± 1.84^d^	23.35 ± 2.06^d^	52.24 ± 2.24^d^
CR3	45.81 ± 3.24^b^	16.38 ± 1.84^b^	43.35 ± 3.87^b^
CR6	43.07 ± 2.42^a^	13.08 ± 2.45^a^	40.81 ± 2.95^a^

The values depict the mean ± SD of three replicate assays. Statistical analysis (One-way ANOVA and Duncan’s multiple range tests) identifies significant differences (*p* ≤ 0.05) marked by superscripts (a–d).

### 3.10. Anti-oxidant activity of LAB isolates

**Figure 8:**
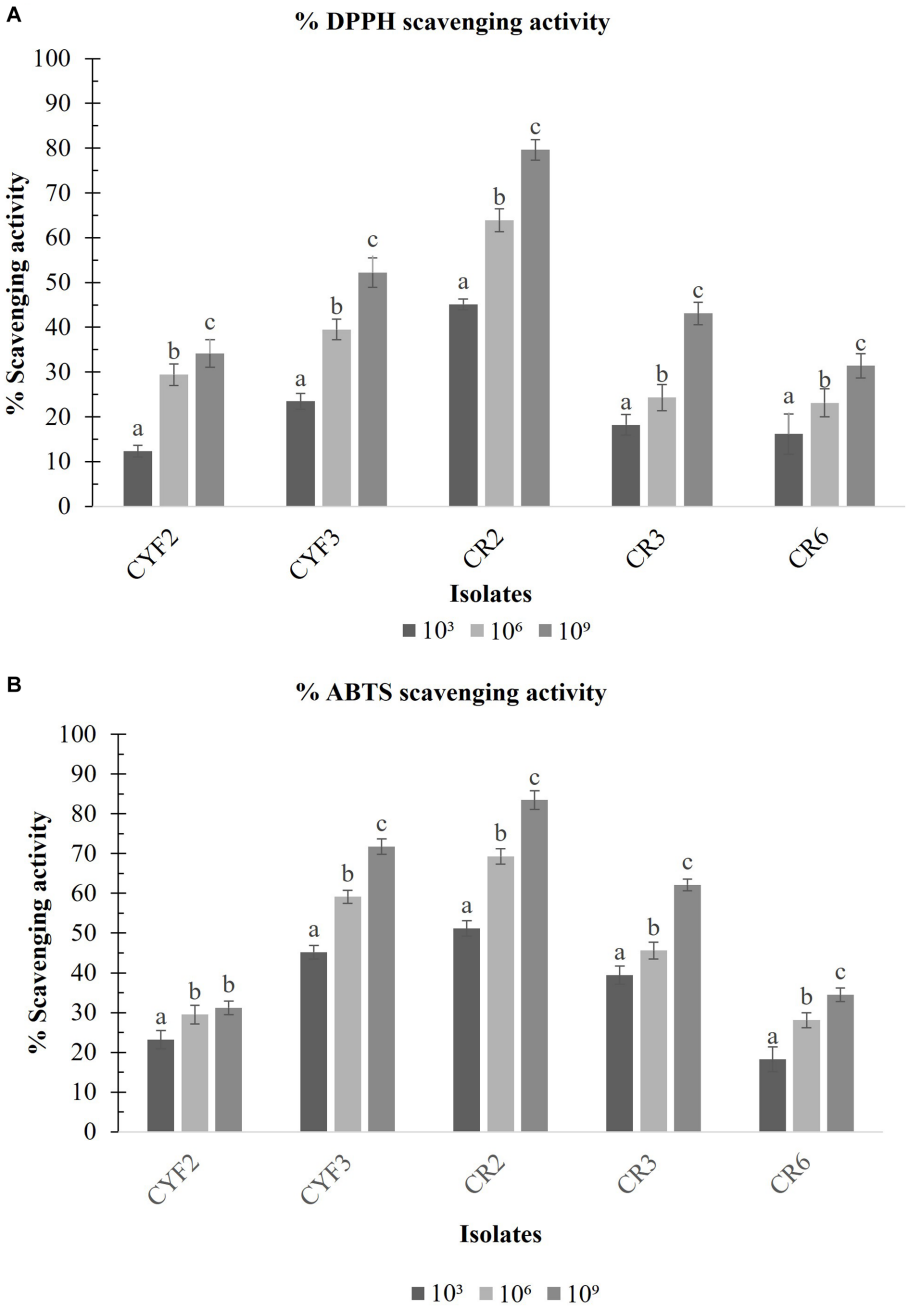
DPPH scavenging activity **(A)** and ABTS scavenging activity **(B)** from five LAB isolates measured in percentage. The values depict the mean ± SD of three replicate assays. Statistical analysis (One-way ANOVA and Duncan’s multiple range tests) identifies significant differences (*p* ≤ 0.05) marked by superscripts (a–c).

With the exponential escalation in cell count, all the isolated specimens exhibited an elevated capacity for DPPH free radical scavenging, notably at a concentration of 10^9^ CFU/mL. The outcomes spanned from a minimal 31.39% for CR6 to a substantial 79.62% for CR2 as depicted in Figure 8A. Similarly, at the same cell concentration of 10^9^ CFU/mL, the range of ABTS radical scavenging activity among the isolates extended from 31.24% for CYF2 to a prominent 83.45% for CR2 as illustrated in Figure 8B.

### 3.11. Assessment of DNase and hemolytic activity

Verifying the safety and non-pathogenic status of a potential probiotic is a critical step. To address this concern, the isolated strains underwent an investigation to assess their DNAse activity. Encouragingly, none of the strains exhibited any DNAse activity, indicating their lack of pathogenic potential and establishing their suitability for consumption. The absence of DNAse activity was consistently confirmed across all selected isolates following a 48-h incubation period at 37°C. Furthermore, a crucial aspect of safety assessment involved the identification of the five LAB isolates based on their γ-hemolytic behavior. This characteristic feature was demonstrated by the absence of discernible zones surrounding the colonies after the incubation period. This absence of hemolysis serves as a strong indicator of the isolates’ safety, thereby enhancing the credibility of their application as a probiotic formulation with an elevated safety profile.

### 3.12. Assessment of antibiotic susceptibility

In accordance with the guidelines proposed by the European Food Safety Authority (EFSA), a comprehensive antibiotic susceptibility test was conducted on the five chosen LAB isolates. Multiple antibiotics were employed to evaluate their susceptibility to different drug classes. All tested LAB isolates demonstrated resistance to Vancomycin and Kanamycin (both 30 μg), as indicated by the phenotypic breakpoints (ZOI ≤ 14). Isolate CR2 displayed resistance to all the antibiotics that were tested, except Chloramphenicol (30 μg), Amoxicillin (10 μg), and Tetracycline (30 μg). Medium sensitivity (ZOI = 15–19 mm) and sensitivity (ZOI ≥ 20 mm) were observed for isolates CR3 and CR6 against most antibiotics. The sensitivity to different antibiotics varied among the isolates, and the results are presented in Table 3.

**Table 3:** Assessment of antibiotic susceptibilities of isolated LAB representing resistance and sensitivity based on CLSI.

Antibiotics	Concentration (μg/ disc)	Isolates from *Theobroma cacao L*.				
		CYF2	CYF3	CR2	CR3	CR6
*Vancomycin*	30	R	R	R	R	R
*Penicillin G*	10	S	I	R	I	S
*Amoxicillin*	10	I	I	I	I	I
*Kanamycin*	30	R	R	R	R	R
*Tetracycline*	30	S	S	I	S	S
*Chloramphenicol*	30	S	I	I	S	I
*Streptomycin*	10	I	S	R	S	S
*Gentamicin*	10	I	S	R	I	S

R, Resistant (Zone size ≤ 14 mm); I, Intermediate (Zone size = 15–19 mm); S, Sensitive (Zone size ≥ 20 mm).

### 3.13. Assessment of antimicrobial activity

The LAB isolates were evaluated for their antimicrobial activity against five pathogens, as summarized in Table 4. Based on their ability to inhibit all tested indicators and exhibit the maximum zone of inhibition (ZOI), five LAB isolates were selected for further investigation of their probiotic properties. These isolates demonstrated notable inhibitory effects against the tested pathogens. Notably, isolate CR2 exhibited the largest inhibition zones against *Escherichia coli, Klebsiella pneumoniae, Pseudomonas aeruginosa, Bacillus cereus*, and *Staphylococcus aureus*, followed by isolate CYF3.

**Table 4:** Assessment of antimicrobial activity.

Isolates	Test organisms (ZOI in mm)				
	*E. coli* MTCC 443	*K. pneumoniae* MTCC 3384	*P. aeruginosa* MTCC 424	*B. cereus* MTCC 430	*S. aureus* MTCC 737
*CYF2*	13.50 ± 0.20^a^	–	–	13.30 ± 0.11^b^	20.50 ± 0.10^b^
*CYF3*	14.30 ± 0.10^b^	18.20 ± 0.20^a^	16.40 ± 0.10^b^	12.06 ± 0.15^a^	22.15 ± 0.07^c^
*CR2*	15.20 ± 0.02^c^	22.23 ± 0.32^b^	19.16 ± 0.25^c^	15.33 ± 0.30^c^	26.63 ± 0.30^e^
*CR3*	–	–	14.20 ± 0.10^a^	13.26 ± 0.15^b^	15.46 ± 0.32^a^
*CR6*	–	–	–	15.50 ± 0.16^c^	25.20 ± 0.08^d^

The value represents the mean zone of inhibition with a standard deviation of three replicates in mm, including a 6.5 mm diameter of the well (symbol ‘–’ shows no zone of inhibition). Statistical analysis (One-way ANOVA and Duncan’s multiple range tests) identifies significant differences (*p* ≤ 0.05) marked by superscripts (a–e).

### 3.14. Molecular characterization of a potential probiotic isolate

**Figure 9:**
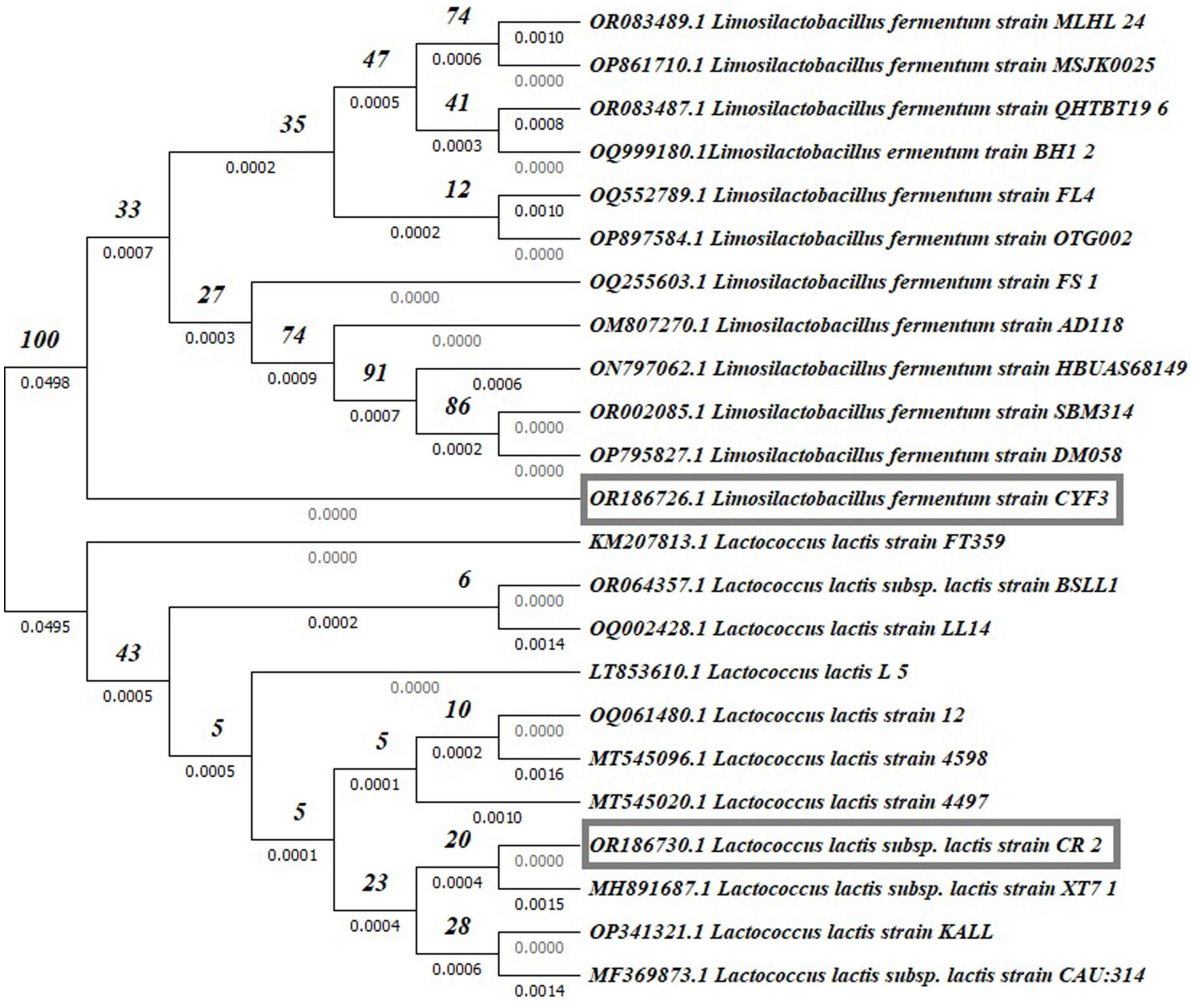
Phylogenetic tree of potent LAB isolates based on neighbor-joining distance analysis of 16S rRNA gene sequences.

Partial 16S rDNA sequencing was systematically conducted to validate and definitively establish the identity of the isolated strains. The phylogenetic placements of these strains within the context of LAB species are depicted in Figure 9. Notably, among these, potential probiotic candidates CR2 and CYF3 were conclusively categorized as *Lactococcus lactis* subsp. *lactis* and *Limnosilactobacillus fermentum*, respectively. This classification was substantiated by achieving 100% sequence similarity with the nearest reference entries in the NCBI GenBank database. Both CR2 and CYF3 exhibited remarkable probiotic and technological attributes, eclipsing the performance of other LAB isolates subjected to scrutiny.

For documentation, the identification and accession codes for the promising LAB isolates were recorded as OR186730 and OR186726 for CR2 and CYF3, respectively. The phylogenetic tree was meticulously constructed using the Neighbor-Joining method (Saitou and Nei, 1987), with the visualization presenting an optimal representation. The associated branches are supplemented with bootstrap replicate percentages (derived from 1000 replicates) to underscore the robustness of taxonomic clustering (Felsenstein, 1985). Evolutionary distances were computed by employing the Maximum Composite Likelihood method (Tamura et al., 2004), expressed in terms of base substitutions per site. The dataset, comprising 23 nucleotide sequences, underwent meticulous curation through pairwise deletion to eliminate ambiguous positions (Tamura et al., 2021), resulting in a final dataset encompassing 1536 positions. The entirety of these sophisticated evolutionary analyses was executed using the MEGA11 software, renowned for its proficiency in such endeavors.

## 4. Discussion

The primary objective of the current investigation was to identify and isolate potentially robust LAB strains with probiotic attributes of Indian origin *Theobroma cacao L*. Among the 11 isolates tested, five exhibited favorable probiotic characteristics. Isolates CR2 and CYF3, identified as *Lactococcus lactis* subsp. *lactis* and *Limnosilactobacillus fermentum* showed strong probiotic potential. *Lactococcus lactis* has been previously identified in cocoa fermentation processes in Ghana (Camu et al., 2007, 2008), the Eastern Region of Ghana (Ayertey et al., 2017), and in Aldama, Tabasco, Mexico (Huerta-Conde et al., 2021). Predominantly, during the intermediate and late stages of cocoa fermentation, the prevailing strictly heterofermentative LAB species have primarily been recognized as *Limosilactobacillus fermentum* (formerly known as *Lactobacillus fermentum*) was well documented in prior research (Camu et al., 2007, 2008; Nielsen et al., 2007; Kostinek et al., 2008; Lefeber et al., 2011; Illeghems et al., 2012, 2015; Moreira et al., 2013, 2017; Papalexandratou et al., 2013, 2019; Pereira et al., 2013a,b; Ho et al., 2014; Ramos et al., 2014; Hamdouche et al., 2015, 2019; Batista et al., 2016; Bortolini et al., 2016; Miescher Schwenninger et al., 2016; Visintin et al., 2016; Miguel et al., 2017; Bastos et al., 2018; Mota-Gutierrez et al., 2018; Oliveira et al., 2018; Romanens et al., 2018; Agyirifo et al., 2019; Lee et al., 2019; Ruggirello et al., 2019; Chagas Junior et al., 2021). Nevertheless, within the scope of our contemporary knowledge, there exists no recorded substantiation of LAB isolation from cacao varieties indigenous to India.

To evaluate potential probiotic candidates, it is essential to consider their ability to withstand highly acidic conditions and maintain a robust bacterial population during the extended 2–3-h transit through the stomach. Additionally, their tolerance to bile salts in the human intestinal environment is a critical factor (Kandylis et al., 2016). The observed decline in isolate growth in our study is attributed to the adverse effects of acidic conditions generated by hydrogen ions (H^+^) on cell walls and metabolic processes (Doyle and Buchanan, 2012). Acid tolerance mechanisms vary by species and strain, explaining differences in isolate tolerance levels. Bile tolerance may be linked to bile deconjugation ability (Liong and Shah, 2005). Notably, *L. lactis* subsp. *lactis* strain CR2 and *Limnosilactobacillus fermentum* strain CYF3 exhibited significant proficiency in both acid and bile tolerance compared to other isolates, consistent with previous studies by El-Jeni et al. (2016), Ayyash et al. (2018), and Srinivash et al. (2023).

The susceptibility of bacteria, particularly Gram-positive types, to bile salts is linked to their direct interaction with cellular membranes. While bile salt efflux mechanisms have been extensively studied in Gram-negative bacteria, some LABs have shown resistance through bile salt hydrolysis (Van de Gutche et al., 2002; Ayyash et al., 2018; dos Santos Leandro et al., 2021). Bile Salt Hydrolase (BSH) specific activity was quantified for sodium glycocholate, sodium taurocholate, and a mixture of bile salts, with values ranging from 0.42 to 2.23 U/mg, 0.30 to 2.86 U/mg, and 0.34 to 3.55 U/mg, respectively. These results align with a previous study by Ayyash et al. (2018), which reported BSH activity ranging from 0.50 to 2.55 U/mg for sodium glycocholate, 0.34 to 2.30 U/mg for sodium taurocholate, and 0.38 to 2.63 U/mg for the mixture of bile salts.

Phenolic conditions, resulting from bacterial deamination of amino acids derived from dietary proteins, are vital for supporting gut microbiota survival. Gut microbes can produce phenol and other potentially harmful metabolites during specific digestive processes, making them potential probiotics (Padmavathi et al., 2018; Huligere et al., 2023). In a study by Huligere et al. (2023), cell viability increased from 7.12 to 7.84 Log CFU/mL after 24 h with a 0.4% phenolic concentration. In our research*, L. lactis* subsp. *lactis* strain CR2 displayed remarkable resilience, with cell viability rising from 7.98 to 8.82 Log CFU/mL under 0.4% phenol exposure, significantly surpassing prior studies. This highlights its effective gastrointestinal transit resilience and superior phenolic tolerance compared to previous findings.

Apart from pH and bile salt challenges, the resilience of probiotics against pepsin and pancreatic enzymes is crucial. We subjected five LAB strains to simulated gastric fluid (pH 2.0, 3 h) followed by simulated intestinal fluid (pH 8.0, 24 h). Notably, *L. lactis* subsp. *lactis* strain CR2 showed a substantial viability shift from 8.23 CFU/mL to 6.60 CFU/mL, yielding an 80.23% survival rate. Hence, the tolerance to environmental challenges varied among strains, in line with prior studies (Gupta and Tiwari, 2015; Tokatli et al., 2015; Kumari et al., 2022; Liu et al., 2022), highlighting strain-specific adaptability.

The ability to resist the effects of lysozyme is a noteworthy probiotic trait, as lysozyme catalyzes the hydrolysis of bacterial cell walls, leading to cell death. Variations in lysozyme resistance among the isolated strains can be attributed to differences in cell wall structures and layers (Doyle and Buchanan, 2012). Our study found that LAB isolates displayed differing lysozyme tolerance levels, resulting in reductions in viable cell counts ranging from 0.23 to 3.80 log CFU/mL. These reductions corresponded to survival rates between 52.98 and 97.19%, consistent with previous research by Yadav et al. (2016), which reported survival rates from 53.45 to 96.69%.

In this study, we conducted a comprehensive safety assessment of the isolated LAB strains, including DNase activity, hemolytic potential, antibiotic susceptibility, and antimicrobial properties. The absence of DNase activity underscores their safety for fermentation processes. Hemolytic assay results confirmed their non-harmful nature, consistent with prior research by Saroj et al. (2016), Ahire et al. (2021), and Srinivash et al. (2023).

Antibiotic susceptibility is critical in assessing probiotic strain safety and addressing antibiotic resistance concerns. In LAB, a common pattern is sensitivity to protein synthesis inhibitors like Tetracycline and Chloramphenicol, while showing resistance to glycopeptides-mediated antibiotics such as Gentamycin, Streptomycin, and Kanamycin. In our study, all five isolates were susceptible to protein synthesis inhibitors, indicating low pathogenic potential and unique isolate characteristics. Considering antibiotic susceptibility, all five isolates were deemed safe and promising probiotic candidates. Assessing safety risks, particularly antibiotic resistance, was a crucial criterion in evaluating these bacteria as probiotics (Naeem et al., 2012; Jampaphaeng et al., 2017; Tarique et al., 2022; Srinivash et al., 2023). Ahire et al. (2021) also emphasized the favorable antibiotic sensitivity of *Lactobacilli* for potential probiotic use.

Co-aggregation serves as a pivotal defensive mechanism, impeding the colonization of pathogenic microorganisms (Choi et al., 2018; Nami et al., 2020; Liu et al., 2022). The capacity of LAB isolates to engage in co-aggregation with pathogens can be attributed to surface-presented protein components and interactions involving carbohydrates and lectins (Nami et al., 2020; Liu et al., 2022). In our study, we evaluated co-aggregation capabilities using pathogenic bacteria, namely, *Escherichia coli* MTCC 443, *Pseudomonas aeruginosa* MTCC 424, and *Staphylococcus aureus* MTCC 737. The results indicated that the propensity for co-aggregation is contingent on the specific combination of LAB strains and pathogenic bacteria, consistent with earlier research findings (Campana et al., 2017; Reuben et al., 2020). Furthermore, we conducted an antagonistic activity test involving *Escherichia coli* MTCC 443, *Klebsiella pneumoniae* MTCC 3384, *Pseudomonas aeruginosa* MTCC 424, *Bacillus cereus* MTCC 430, and *Staphylococcus aureus* MTCC 73 bacteria. Notably, the results highlighted that the *L. lactis* subsp. *lactis* strain CR2 isolate, followed by *Limnosilactobacillus fermentum* strain CYF3, demonstrated significant antibacterial efficacy against all enteric pathogens.

Hydrophobicity and auto-aggregation are crucial factors for LAB colonization in the intestinal wall. Previous research (Liu et al., 2022) showed significant cell surface hydrophobicity (>60%) and notable auto-aggregation (>40%) in *Lactobacillus* spp. In our study, all five isolates exhibited robust cell surface hydrophobicity, ranging from 50.45 to 95.00% with chloroform and 54.13 to 78.28% with xylene. They also demonstrated elevated auto-aggregation levels, ranging from 50.29 to 82.96%. These findings emphasize their potential for effective colonization, consistent with prior research (Somashekaraiah et al., 2019; Li et al., 2020; Reuben et al., 2020; Jeong et al., 2021).

The evaluation of α-glucosidase enzyme activity provides insights into the potential to inhibit glucose production and gradually reduce postprandial hyperglycemic blood glucose absorption in the small intestine (Ramu et al., 2014; Martiz et al., 2022). The strain TKSP 24, from Korean fermented soybean sauce (Doenjang), displayed α-glucosidase inhibitory activity ranging from 58 to 62% (Shukla et al., 2016). In our investigation, the LAB isolates exhibited a substantial 56.55% inhibition potential in the Cell-Free Supernatant (CS). LAB isolate *L. lactis* subsp. *lactis* strain CR2 showed an inhibitory potential toward α-glucosidase similar to prior studies.

In the current study, probiotic LAB strains have demonstrated strong proficiency in EPS production, which finds diverse applications in the food industry such as stabilization, emulsification, texturization, syneresis reduction, viscosity enhancement, and water-binding capabilities. Additionally, their pseudoplastic rheological behavior makes them suitable for improving the sensory qualities of dairy products. EPS synthesis by LAB offers health benefits including blood pressure regulation, cholesterol reduction (Shivangi et al., 2020), immune stimulation, and potential antitumor activities (Roux et al., 2022). Srinivash et al. (2023) studied LAB strains for EPS production, highlighting *L. delbrueckii* strain GRIPUMSK with the highest production at 0.69 mg/mL. In comparison, *L. lactis* subsp. *lactis* strain CR2 from *Theobroma cacao L.* displayed EPS production of 0.66 mg/mL, aligning with the earlier study production levels.

Bacterial cell surface constituents play a crucial role in countering the harmful effects of free radicals. Our study found that the investigated isolates displayed a high capacity for scavenging free radicals, consistent with prior research. Free radicals are implicated in diabetes onset and progression (Alkalbani et al., 2019; Kim et al., 2019), with hydroxyl and similar radicals particularly damaging to biomolecules. Antioxidants stabilize molecules by donating electrons or hydrogen atoms, as seen in DPPH and ABTS assays. The DPPH free radical scavenging activity at a concentration of 10^9^ CFU/mL ranged from 26.12 to 76.63%, while the ABTS radical scavenging abilities spanned from 31.15 to 84.45% in accordance with Huligere et al. (2023). Our observations confirmed these trends, with DPPH free radical scavenging potential ranging from 31.39 to 79.62% and ABTS radical scavenging efficacy from 31.24 to 83.45%, consistent with previous results.

## 5. Conclusion

The escalating prevalence of multidrug resistance among pathogens has led to increased interest in probiotics within the food industry. This has prompted a demand for conventional or indigenous probiotics as alternatives to long-lasting chemotherapeutic treatments. In this study, our focus was on expanding the selection of probiotic bacteria from fermented fruit, specifically *Theobroma cacao L*. Based on the obtained results, it can be concluded that the isolates *Lactococcus lactis* subsp. *lactis* and *Limnosilactobacillus fermentum* demonstrated promising probiotic potential. Therefore, it could be utilized as a starter culture for the production of probiotic-based food products. However, further *in vivo* investigations are necessary to validate its potential health benefits and explore its broader applications.

## Data availability statement

The datasets presented in this study can be found in online repositories. The names of the repository/repositories and accession number(s) can be found at: https://www.ncbi.nlm.nih.gov/, OR186730; https://www.ncbi.nlm.nih.gov/, OR186726.

## Author contributions

MN: Conceptualization, Investigation, Writing – original draft. RS: Supervision, Writing – review & editing.

## Funding

The author(s) declare that no financial support was received for the research, authorship, and/or publication of this article.

## Conflict of interest

The authors declare that the research was conducted in the absence of any commercial or financial relationships that could be construed as a potential conflict of interest.

## Publisher’s note

All claims expressed in this article are solely those of the authors and do not necessarily represent those of their affiliated organizations, or those of the publisher, the editors and the reviewers. Any product that may be evaluated in this article, or claim that may be made by its manufacturer, is not guaranteed or endorsed by the publisher.
