# Supplementary material for: Exploration of probiotic attributes in lactic acid bacteria isolated from fermented Theobroma cacao L. fruit using in vitro techniques
Source: Front Microbiol. 2023 Sep 21;14:1274636. doi: 10.3389/fmicb.2023.1274636 (PMC10552159; doi:10.3389/fmicb.2023.1274636)
Supplement: Supplementary file 1 [file Data_Sheet_1.docx]

**TABLE 1. Physiological and biochemical properties of isolates**

| **Parameters** | **Isolates from *Theobroma cacao L.*** | | | | |
| --- | --- | --- | --- | --- | --- |
|  | **CYF2** | **CYF3** | **CR2** | **CR3** | **CR6** |
| **Gram reaction and shape** | +ve, rods | +ve, coccus | +ve, coccos | +ve, rods | +ve, coccoid/ rod |
| **Endospore Formation test** | - | - | - | - | - |
| **Motility test** | - | - | - | - | - |
| **Biochemical characters** | | | | | |
| **Catalase test** | - | - | - | - | - |
| **Oxidase test** | - | - | - | - | - |
| **Citrate utilization** | - | - | - | - | - |
| **Methyl red test** | - | - | - | - | - |
| **Voges Prausker test** | - | - | - | - | - |
| **Iodine test** | - | - | - | - | - |
| **Carbohydrate fermentation** | | | | | |
| **Glucose** | + | + | + | + | + |
| **Fructose** | + | + | + | + | + |
| **Ribose** | + | + | + | + | + |
| **Galactose** | + | + | + | + | + |
| **Mannitol** | + | + | + | + | + |
| **Xylose** | + | + | + | + | + |
| **Maltose** | + | + | + | + | + |
| **Lactose** | + | + | + | + | + |
| **Sucrose** | + | + | + | + | + |

+= Positive test, - = Negative test

**Antimicrobial activity of LAB isolated from Theobroma cacao L.**
